# Phosphine Sulfide-Based Bipolar Host Materials for Blue Phosphorescent Organic Light-Emitting Diodes

**DOI:** 10.3390/molecules26134079

**Published:** 2021-07-03

**Authors:** Gaozhan Xie, Jiangchao Wang, Yang Cao, Xudong Xue, Xiao Zhang, Chang Liu, Huanhuan Li, Ye Tao, Runfeng Chen

**Affiliations:** State Key Laboratory of Organic Electronics and Information Displays & Institute of Advanced Materials (IAM), Jiangsu National Synergistic Innovation Center for Advanced Materials (SICAM), Nanjing University of Posts & Telecommunications, 9 Wenyuan Road, Nanjing 210023, China; iamgzxie@njupt.edu.cn (G.X.); 1220066216@njupt.edu.cn (J.W.); 1019061401@njupt.edu.cn (Y.C.); 1020062012@njupt.edu.cn (X.X.); 18805197992@163.com (X.Z.); 1020061916@njupt.edu.cn (C.L.); iamrfchen@njupt.edu.cn (R.C.)

**Keywords:** bipolar host material, phosphorescent organic light-emitting diode, phosphine sulfide, carbazole, triplet energy level

## Abstract

Three phosphine sulfide-based bipolar host materials, *viz* **CzPhPS**, **DCzPhPS**, and **TCzPhPS**, were facilely prepared through a one-pot synthesis in excellent yields. The developed hosts exhibit superior thermal stabilities with the decomposition temperatures (*T*_d_) all exceeding 350 °C and the melting temperatures (*T*_m_) over 200 °C. In addition, their triplet energy (*E*_T_) levels are estimated to be higher than 3.0 eV, illustrating that they are applicable to serve as hosts for blue phosphorescent organic light-emitting diodes (PhOLEDs). The maxima luminance, current efficiency (CE), power efficiency (PE), and external quantum efficiency (EQE) of 17,223 cd m^−2^, 36.7 cd A^−1^, 37.5 lm W^−1^, and 17.5% are achieved for the blue PhOLEDs hosted by **CzPhPS**. The PhOLEDs based on **DCzPhPS** and **TCzPhPS** show inferior device performance than that of **CzPhPS**, which might be ascribed to the deteriorated charge transporting balance as the increased number of the constructed carbazole units in **DCzPhPS** and **TCzPhPS** molecules would enhance the hole-transporting ability of the devices to a large extent. Our study demonstrates that the bipolar hosts derived from phosphine sulfide have enormous potential applications in blue PhOLEDs, and the quantity of donors should be well controlled to exploit highly efficient phosphine sulfide-based hosts.

## 1. Introduction

Phosphorescent organic light-emitting diodes (PhOLEDs) have been extensively researched in recent decades since they can achieve an internal quantum efficiency of nearly 100% by harvesting both singlet and triplet excitons, and have the advantages of excellent device stability, high color purity, as well as flexible color adjustment [1,2,3]. Nevertheless, the emitters in PhOLEDs need to be incorporated into appropriate hosts for the reason that the high concentration of emitters can result in concentration quenching, such as triplet–triplet annihilation (TTA) and triplet–singlet annihilation (TSA) [4,5]. As a consequence, it is of extreme importance to exploit the state-of-the-art hosts to attain high-performance PhOLEDs.

Bipolar hosts, generally prepared by combining the donor (D) and acceptor (A) units into a molecular skeleton (i.e., D–A molecules), are capable of achieving a broad recombination zone and balanced charge in the emitting layer (EML), so they are considered one of the most promising types of hosts to realize highly efficient PhOLEDs [6,7,8]. Up until the present, the bipolar hosts for green, yellow, and red phosphorescent emitters have made commendable progress [9,10,11,12], while it is still challenging to develop superior blue hosts because the blue phosphors inherently possess high triplet energy (*E*_T_) levels [13,14,15], thus the *E*_T_ of the hosts should be accordingly elevated to prevent the reverse energy transfer from the guests back to the hosts and to effectively confine triplet excitons on the guests. However, the non-negligible intramolecular interactions in D–A molecules would produce a low energy charge transfer (CT) state, which brings down the *E*_T_ levels [16]. Therefore, for the design of blue bipolar hosts that exhibit the balanced charge transport ability simultaneously maintaining a high *E*_T_, D and A constructed groups should be meticulously selected.

**Scheme 1:**
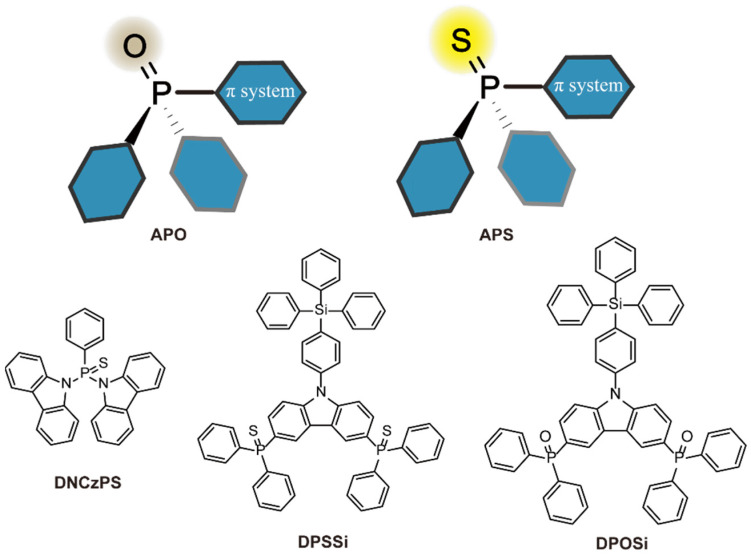
Chemical structures of APO, APS, **DNCzPS**, **DPSSi**, and **DPOSi**.

Aryl phosphine oxide (APO, Scheme 1), with moderated electron-withdrawing ability and excellent charge transport properties, has been widely applied in blue bipolar hosts [17,18,19,20,21,22,23,24]. Through simply replacing the O atom of APO with the S atom, aryl phosphine sulfide (APS, Scheme 1) can be gained. Since the ^3^*p* lone-pair electrons of S exhibit the larger scope than the ^2^*p* lone-pair electrons of O, the sulfur atom and π–system interactions (S–π interactions) can be formed, which would partly enhance intramolecular electronic communication, leading to selectively modulated frontier molecular orbital energy levels and improved electrical performance [25,26]. On the other hand, APO with stronger polarization of P=O exhibits weaker intramolecular electronic coupling and an unduly deep highest occupied molecular orbital (HOMO) energy level, rendering it difficult to maintain the efficient and balanced charge injection and transportation. Hence, the hosts consisting of APS tend to have enhanced electrical performance than that of APO. However, the APS-based hosts have hardly been reported so far, and need to be further exploited. In 2016, our group reported a variety of remarkably high-performance bipolar hosts adopting the phenylphosphine sulfide as the electron-withdrawing unit, of which the host named as **DNCzPS** ((9*H*-carbazol-9-yl)diphenylphosphine sulfide, Scheme 1) displays a *E*_T_ level as high as 2.97 eV [25]. The blue PhOLED based on **DNCzPS** turns on at 2.9 eV with an impressive maximum external quantum efficiency (EQE) of 21.7%. Subsequently, we prepared the APS-based host **DPSSi** ((9-(4-(triphenylsilyl)phenyl)-9*H*-carbazole-3,6-diyl) bis(diphenyl-phosphine sulfide), Scheme 1), whose blue PhOLED shows a maximum EQE of 20.8%, which is 1.58 folds of enhancement compared to that of the APO-based host **DPOSi** ((9-(4-(triphenylsilyl)phenyl)-9*H*-carbazole-3,6-diyl)bis(diphenylphosphine oxide), Scheme 1) in the same device structure [26]. Encouraged by the above-mentioned results, in this contribution we employ triphenylphosphine sulfide as A group, and select mono-, di-, as well as tri-substituted carbazoles with tunable hole-transport ability as D units, respectively, to construct three original bipolar hosts, i.e., **CzPhPS**, **DCzPhPS**, and **TCzPhPS**. All three compounds possess the high *E*_T_s of ~3.0 eV, much higher than those of the commonly used blue emitters, demonstrating that they are promising candidates as the hosts for blue OLEDs. The blue PhOLED hosted by **CzPhPS** achieves a low turn-on voltage of 3.1 eV and a maximum EQE of 17.5%, better than the device performance of **DCzPhPS** and **TCzPhPS,** which could be owed to its better charge transport balance.

## 2. Results and Discussion

### 2.1. Material Synthese

**Scheme 2:**
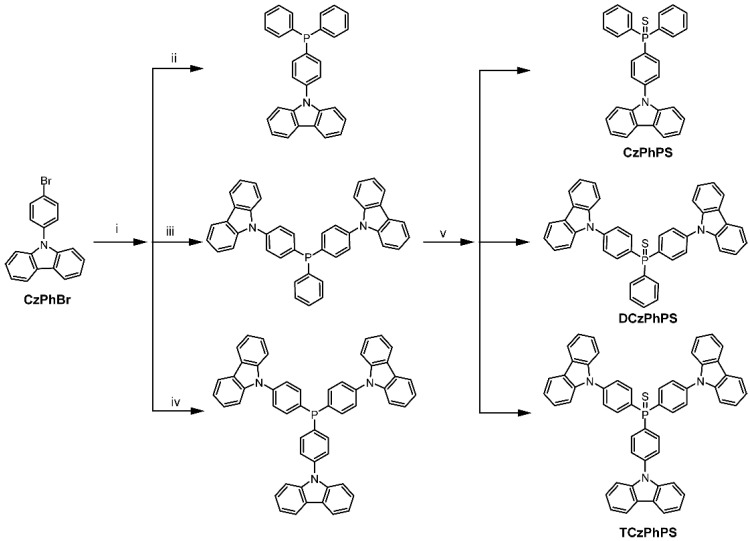
Synthetic routes to **CzPhPS**, **DCzPhPS**, and **TCzPhPS**. (i) *n*-BuLi, THF, −78 °C, 2 h; (ii) Ph_2_PCl, −78 °C, 1 h; (iii) PhPCl_2_, −78 °C, 1 h; (iv) P(OCH_2_CH_3_)_3_, −78 °C, 1 h; (v) Sulfur, CH_2_Cl_2_, room temperature (r.t.), overnight.

**CzPhPS**, **DCzPhPS**, and **TCzPhPS** were facilely obtained by a one-pot synthesis as outlined in Scheme 2. A nucleophilic addition reaction of the organolithium compounds formed from 9-(4-bromophenyl)-9*H*-carbazole and *n*-BuLi followed by the treatment with sublimed sulfur in dichloromethane furnished the three target molecules in 57–63% yields. Their molecular structures were firmly confirmed by ^1^H and ^13^C nuclear magnetic resonance (NMR) spectra, as well as high-resolution mass spectra (HRMS) (Supporting Information, Figures S1–S9).

### 2.2. Thermal Stability

**Figure 1:**
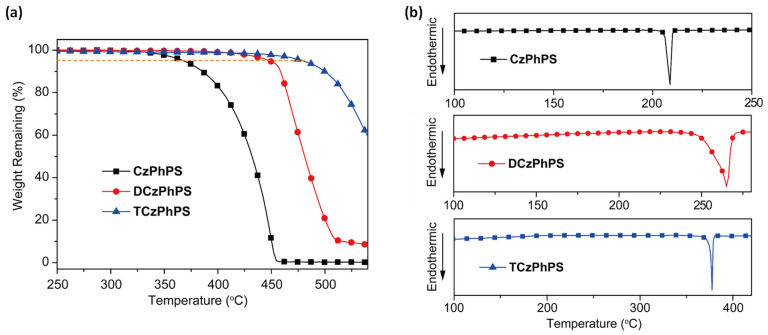
TGA (**a**) and DSC (**b**) curves of **CzPhPS**, **DCzPhPS**, and **TCzPhPS**.

In order to investigate the thermal properties of the designed phosphine sulfide molecules, the thermal gravimetric analysis (TGA) and differential scanning calorimetry (DSC) measurements were carried out under a nitrogen atmosphere. As shown in Figure 1a and Table 1, all three compounds exhibit outstanding thermal stability with the decomposition temperatures (*T*_d_) of 366, 447, and 479 °C for **CzPhPS**, **DCzPhPS**, and **TCzPhPS,** respectively, indicating that P=S bonds are stable enough for the evaporation process to fabricate OLEDs. In addition, for these compounds, no glass transition temperature (*T*_g_) is detected in the tested temperature range shown in Figure 1b, while their melting temperatures (*T*_m_) can be clearly observed with the *T*_m_ values all exceeding 200 °C. From **CzPhPS** to **TCzPhPS**, as expected, the phase stability becomes better due to the increased number of carbazole units.

### 2.3. Photophysical Properties

**Figure 2:**
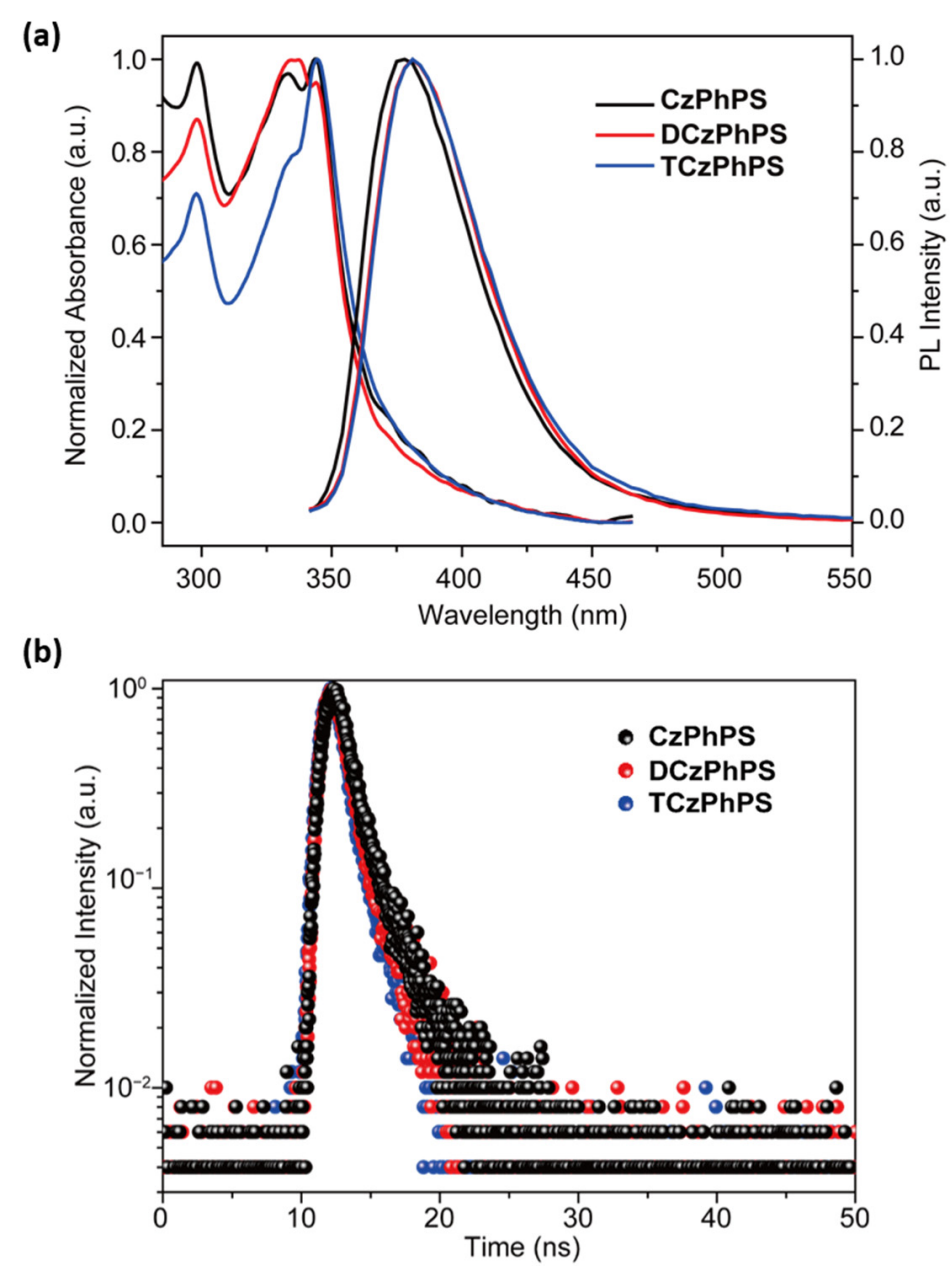
Absorption and PL spectra (**a**), and time-resolved transient PL decay curves (**b**) of **CzPhPS**, **DCzPhPS**, and **TCzPhPS** in solid thin films.

Figure 2a displays the absorption and photoluminescence (PL) spectra of the developed materials in solid thin films. **CzPhPS**, **DCzPhPS**, and **TCzPhPS** exhibit quite similar absorption and emission spectra as they are constructed by the same electron-accepting unit (triphenyl phosphine sulfide) and electron-donating unit (carbazole), and the quantities of carbazole units do not have a large effect on their intermolecular charge transfer properties. All three compounds exhibit a sharp absorption peak at 298 nm, which can be ascribed to the π-π* transitions of carbazole groups [27], whereas the absorption peaks of around 335 and 343 nm might be due to the intramolecular CT from the electron donor carbazole to the electron acceptor triphenyl phosphine sulfide. **CzPhPS**, **DCzPhPS**, and **TCzPhPS** display similar PL spectra with the emission peaks at 378, 381, and 380 nm, respectively. In addition, all the time-resolved transient PL decay curves of those pure films shown in Figure 2b consist of a nanosecond-order lifetime of ~2 ns, suggesting their fluorescent nature. To further investigate the luminescent properties of the target molecules, their photoluminescence quantum yields (PLQYs) were also measured in 10^−5^ M dichloromethane solution. **CzPhPS**, **DCzPhPS**, and **TCzPhPS** exhibit low PLQYs of 24%, 20%, and 22%, respectively. Moreover, according to the first peaks of phosphorescence spectra (see Figure S10) recorded at 77 K in THF with a delay time of 20 ms, the *E*_T_s of **CzPhPS**, **DCzPhPS**, and **TCzPhPS** are estimated to be ~3.0 eV, which is high enough for them to serve as hosts for commonly used blue emitters such as bis(3,5-difluoro-2-(2-pyridyl)phenyl-(2-carboxypyridyl)iridium (III) (FIrpic, *E*_T_: 2.65 eV) [28].

### 2.4. Electrochemical Properties

**Figure 3:**
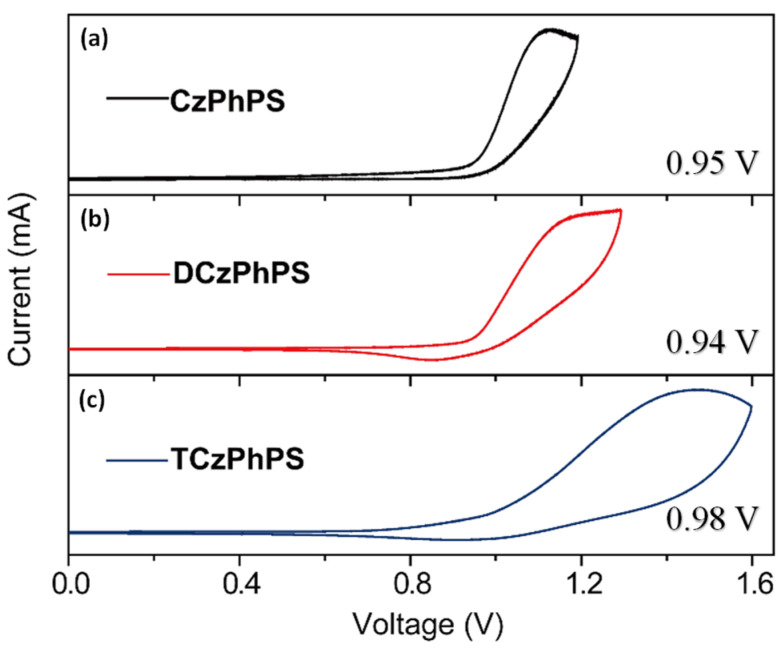
Cyclic voltammograms of (**a**) **CzPhPS**, (**b**) **DCzPhPS**, and (**c**) **TCzPhPS** films deposited on the working electrodes.

Cyclic voltammetry (CV) measurements were carried out to obtain the electrical properties of these phosphine sulfide compounds [29]. As shown in Figure 3, all the compounds exhibit an irreversible oxidative wave with the onset potential at ~0.95 eV, attributed to the oxidation of carbazole units, whereas their reduced waves can not be observed when the negative voltage is applied. The HOMO energy levels are estimated to be ~−5.72 eV based on the onset potential of the oxidative wave, and their lowest unoccupied molecular orbital (LUMO) energy levels are calculated to be −2.29, −2.28, and −2.34 eV for **CzPhPS**, **DCzPhPS**, and **TCzPhPS,** respectively, according to the HOMO energy levels and the absorption edge of thin films.

### 2.5. Theoretical Investigations

**Figure 4:**
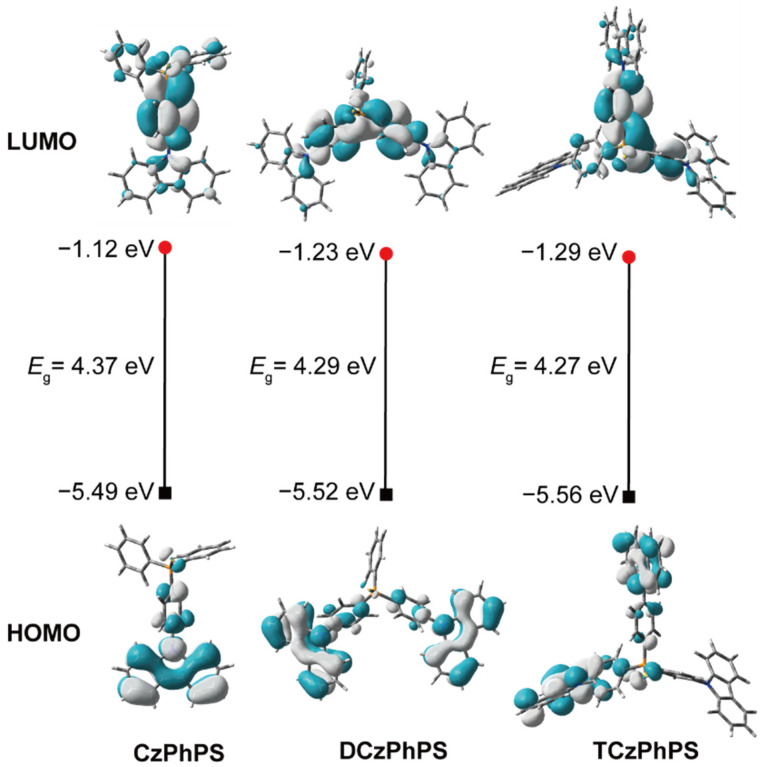
HOMO and LUMO distributions of the developed molecules calculated at the TD-DFT/M062X level by Gaussian 09W.

Density-functional theory (DFT) calculations were further performed to acquire insight into the structure–property relationships of **CzPhPS**, **DCzPhPS**, and **TCzPhPS [30]**. As shown in Figure 4, the LUMO of these three compounds is predominantly located on the triphenylphosphine sulfide group due to its electron-withdrawing feature, while the HOMO is mainly distributed on electron-donating carbazole units, as expected. The separated HOMO and LUMO distributions indicate that the developed compounds should be of bipolar charge carrier transporting ability, which are suitable for being served as bipolar hosts in OLEDs. In addition, the HOMO/LUMO energy levels are simulated to be −5.49/−1.12 eV, −5.52/−1.23 eV, and −5.56/−1.29 eV for **CzPhPS**, **DCzPhPS**, and **TCzPhPS,** respectively, calculated at the TD-DFT/M062X level using Gaussian 09W software. The trend is fairly in line with the above-mentioned CV measurements. In addition, the computational *E*_T_ levels are almost the same with experimental results evaluated from phosphorescent spectra with the values of ~3.0 eV.

### 2.6. Device Fabrication and Performance

**Figure 5:**
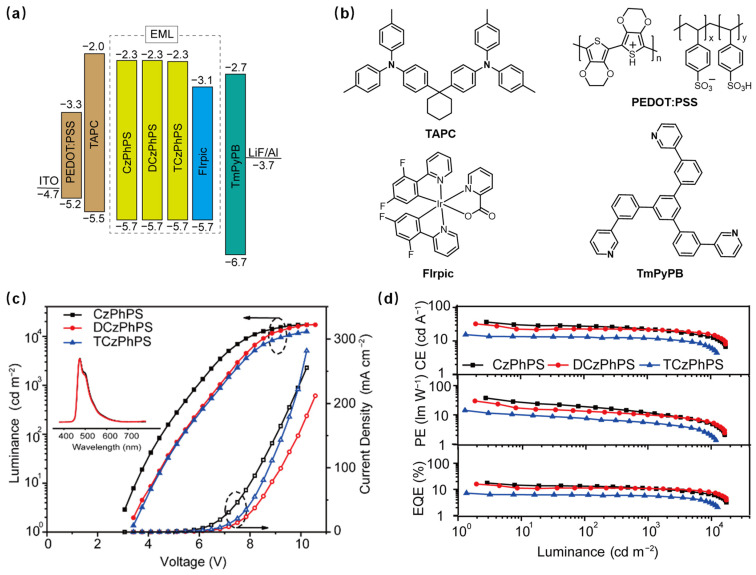
Energy level diagrams (**a**) and chemical structures (**b**) of the materials used in the devices. Luminance-voltage-current density (**c**) and efficiencies-luminance (**d**) curves of the blue PhOLEDs devices. Inset: EL spectra at 5 V.

In light of the high *E*_T_ energy levels and excellent bipolar charge transporting ability of the developed phosphine sulfide materials, the blue PhOLEDs hosted by **CzPhPS** (Device A), **DCzPhPS** (Device B), and **TCzPhPS** (Device C) were fabricated in a typical architecture of indium tin oxide (ITO)/poly(3,4-ethylenedioxythiophene):poly(styrenesulfonate) (PEDOT:PSS) (30 nm)/1,3,5-triazo-2,4,6-triphosphorine-2,2,4,4,6,6-tetrachloride (TAPC) (20 nm)/host: 25 wt% FIrpic (20 nm)/1,3,5-tri(*m*-pyrid-3-yl-phenyl) benzene (TmPyPB) (35 nm)/lithium fluoride (LiF) (1 nm)/Al (100 nm). The energy level diagrams and chemical structures of the adopted materials in the blue PhOLEDs are displayed in Figure 5a,b. Given that the HOMO levels of all three host materials are just 0.2 eV deeper-lying than that of TAPC, there should be no hole-injection barrier between HTL and EML [31]. Likewise, there should be no problem for electrons to jump from ETL to EML as their LUMO energy gap is only 0.4 eV. Luminance–voltage–current density characteristics, EL spectra, and efficiencies–luminance curves of the devices are shown in Figure 5c,d; the corresponding data are listed in Table 2. Device A turns on at a low voltage of 3.1 eV with a peak wavelength, maxima luminance, current efficiency (CE), power efficiency (PE), and EQE of 472 nm, 17,223 cd m^−2^, 36.7 cd A^−1^, 37.5 lm W^−1^, and 17.5%. Notably, Device B and Device C exhibit inferior device performance with maxima EQE of 16.1% and 7.2%, respectively. It is well known that carrier transporting balance is of particular importance to render the holes, and electrons recombine in the emitting layer so that high-performance OLEDs can be achieved [32]. In addition, introducing more carbazole groups into molecule skeletons would increase the hole-transporting ability of the compounds [33,34,35]. Hence, it is rational to deduce that the poorer device performances of Device B and Device C might be ascribed to the increased number of carbazoles in the host molecules, which enhance the hole-transporting ability of the devices, thus deteriorating the carrier transporting balance.

## 3. Materials and Methods

### 3.1. General Methods

^1^H and ^13^C-NMR spectra were recorded on a Bruker Ultra Shield Plus 400 MHz instrument using CDCl_3_ as the solvent and tetramethylsilane (TMS) as the internal standard. The quoted chemical shifts are in *ppm* and the *J* values are expressed in Hz. The splitting patterns have been designed as follows: s (singlet), d (doublet), t (triplet), dd (doublet of doublets), and m (multiplet). HRMS were recorded on a LCT Premier XE (Waters) mass spectrometer. TGA measurements were conducted on a Netzsch STA2500 thermogravimetric analyzer at a heating rate of 10 °C min^−1^ and a nitrogen flow rate of 50 cm^3^ min^−1^. DSC analyses were performed on a Netzsch DSC214 Polyma instrument under a heating rate of 10 °C min^−1^ and a nitrogen flow rate of 20 cm^3^ min^−1^. Ultraviolet-visible (UV-Vis) and fluorescence spectra were recorded on a Jasco V-750 spectrophotometer and Edinburgh FLS980, respectively. The thin films for UV-Vis spectroscopy measurements were prepared by high-vacuum thermal evaporation at a rate of 0.1–0.2 nm s^−1^. The phosphorescence spectra of the compounds in CH_2_Cl_2_ were measured using a time-resolved Edinburgh FLS980 fluorescence spectrophotometer at 77 K, with a 20 ms delay time using a microsecond flash lamp. CV measurements were performed at room temperature on a CHI660E system in a typical three-electrode cell with a working electrode (glass carbon), a reference electrode (Ag/Ag^+^, referenced against ferrocene/ferrocenium (FOC)), and a counter electrode (Pt wire) in an acetonitrile solution of tetrabutylammonium hexafluorophosphate (Bu_4_NPF_6_) (0.1 M) at a sweeping rate of 100 mV s^−1^. The thin solid films of the developed molecules for CV measurements were formed by adding their solution dropwise on the surface of the glassy carbon working electrode followed by drying under ambient conditions. The HOMO energy levels (*E*_HOMO_) of the materials were measured according to the reference energy level of ferrocene (4.8 eV below the vacuum) as illustrated in Equation (1):

(1) $$E_{\mathrm{HOMO}}=-\left[E_{\mathrm{onset}}^{\mathrm{ox}}-E_{\left(\mathrm{Fc}/{\mathrm{Fc}}^{+}\right)}+4.8\right]\;\mathrm{eV}$$

where $E_{\left(\mathrm{Fc}/{\mathrm{Fc}}^{+}\right)}$ is the onset oxidative voltage of FOC vs. Ag/Ag^+^ whose value is 0.4 V in our testing conditions, and $E_{\mathrm{onset}}^{\mathrm{ox}}$ is the onset potential of the oxidation wave. The LUMO energy level (*E*_LUMO_) was estimated by adding the optical band-gap (*E*_g_) to the corresponding HOMO energy level as in Equation (2):

(2) $$E_{\mathrm{LUMO}}=-\left[E_{\mathrm{HOMO}}-E_{g}\right]\;\mathrm{eV}$$

### 3.2. Material Syntheses

The manipulations involving air-sensitive reagents were performed in an atmosphere of dry Argon (Ar). The chemicals and solvents, unless otherwise specified, were purchased from Aladdin, Aldrich or Acros, and used without further purification.

#### 3.2.1. Synthesis of (4-(9*H*-Carbazol-9-yl)phenyl)diphenylphosphine Sulfide (**CzPhPS**)

9-(4-Bromophenyl)-9*H*-carbazole (3.2 g, 10.0 mmol, 1.0 equivalent (eq.)) was dissolved in 50 mL anhydrous THF under Ar and cooled to −78 °C in a dry ice/acetone bath. *n*-BuLi (7.5 mL, 12.0 mmol, 1.2 eq., 1.6 M in hexane) was then added dropwise to give a yellow solution. Two hours later, 2.0 mL diphenylphosphine chloride (10.0 mmol, 1.0 eq.) was injected into the system at −78 °C and the mixture was stirred at this temperature for 1 h, following which the temperature was allowed to increase to ambient. After stirring overnight, the reaction was quenched with 50 mL water and extracted by dichloromethane (3 × 50 mL). The organic layer was dried with anhydride Na_2_SO_4_, and the solvent was evaporated under reduced pressure. The resulting solid resides were dissolved in dichloromethane (50 mL) for sulfurization by treating with an excessive amount of sulfur (1.0 g, 30 mmol, 3.0 eq.). After stirring overnight at room temperature, the solvent was removed, and the crude product was purified by a column chromatography to obtain a pure white solid. Yield: 2.9 g, 63.0%. ^1^H NMR (400 MHz, CDCl_3_, ppm) δ 8.15 (d, *J* = 8.0 Hz, 2H), 7.98 (dd, *J* = 12.0, 12.0 Hz, 2H), 7.86 (m, 4H), 7.71 (dd, *J* = 8.0, 4.0 Hz, 2H), 7.57 (m, 8H), 7.44 (m, 2H), 7.33 (m, 2H). ^13^C NMR (100 MHz, CDCl_3_, ppm) δ 140.94, 140.19, 134.03, 133.92, 133.05, 132.39, 132.28, 132.20, 132.13, 131.83, 131.81, 131.28, 128.78, 128.66, 126.52, 126.39, 126.18, 123.81, 120.61, 120.47, 109.77. HRMS (EI): *m*/*z* calcd for C_30_H_22_NPS [M + H]^+^: 460.1289; found: 460.1284.

#### 3.2.2. Synthesis of Bis(4-(9*H*-carbazol-9-yl)phenyl)(phenyl)phosphine Sulfide (**DCzPhPS**)

**DCzPhPS** was prepared under the identical synthetic conditions described in the preparation of **CzPhPS** using 9-(4-bromophenyl)-9*H*-carbazole (3.2 g, 10.0 mmol, 2.0 eq.), *n*-BuLi (7.5 mL, 12 mmol, 2.4 eq., 1.6 M in hexane), phenylphosphane dichloride (0.7 mL, 5.0 mmol, 1.0 eq.) and sulfur (0.5 g, 15 mmol, 3.0 eq.). Yield: 1.8 g of white power (57.6%). ^1^H NMR (400 MHz, CDCl_3_, ppm) δ 8.16 (d, *J* = 8.0 Hz, 4H), 8.07 (m, 4H), 7.97 (m, 2H), 7.79 (dd, *J* = 4.0 Hz, 4H), 7.62 (m, 3H), 7.55 (d, *J* = 8.0 Hz, 4H), 7.46 (m, 4H), 7.35 (m, 4H). ^13^C NMR (100 MHz, CDCl_3_) δ 141.17, 140.17, 134.08, 133.97, 132.43, 132.33, 132.05, 131.75, 130.89, 128.96, 128.84, 126.65, 126.52, 126.22, 123.87, 120.69, 120.51, 109.78. HRMS (EI): *m*/*z* calcd for C_42_H_29_N_2_PS [M + H]^+^: 625.1867; found: 625.1865.

#### 3.2.3. Synthesis of Tris(4-(9*H*-carbazol-9-yl)phenyl)phosphine Sulfide (**TCzPhPS**)

**TCzPhPS** was prepared under the identical synthetic conditions described in the preparation of **CzPhPS** using 9-(4-bromophenyl)-9*H*-carbazole (2.4 g, 7.5 mmol, 3.0 eq.), *n*-BuLi (5.0 mL, 8.0 mmol, 3.2 eq.,1.6 M in hexane), triethyl phosphite (0.43 mL, 2.5 mmol, 1.0 eq.) and sulfur (0.25 g, 7.5 mmol, 3.0 eq.). Yield: 1.2 g of white power (60.8%). ^1^H NMR (400 MHz, CDCl_3_, ppm) δ 8.20 (m, 12H), 7.86 (m, 6H), 7.59 (d, *J* = 8.0 Hz, 6H), 7.47 (m, 6H), 7.36 (t, *J* = 8.0 Hz, 6H); ^13^C NMR (100 MHz, CDCl_3_, ppm) δ 141.46, 140.15, 134.13, 131.39, 130.52, 126.78, 126.27, 123.93, 120.78, 109.78; HRMS (EI): *m*/*z* calcd for C_54_H_36_N_3_PS [M + H]^+^: 790.2446; found: 790.2448.

### 3.3. Device Fabrication and Characterization

Typically, ITO-coated glass substrates were etched, patterned, and washed by ultrasonic cleaner with detergent, deionized water, acetone, and ethanol in turn. Organic layers were deposited by high-vacuum (10^−6^ Torr) thermal evaporation at a rate of 0.1–0.2 nm s^−1^. The layer thickness and the deposition rate were monitored in situ by an oscillating quartz thickness monitor. The devices were measured after fabrication without encapsulation under an ambient atmosphere at room temperature. EL spectra of the devices were measured by a PR655 spectra scan spectrometer. The luminance–voltage and current–voltage characteristics were recorded using an optical power meter and a Keithley 2602 voltage current source.

## 4. Conclusions

In conclusion, we successfully synthesized three novel bipolar hosts, **CzPhPS**, **DCzPhPS**, and **TCzPhPS,** by using triphenylphosphine sulfide as an electron-deficient unit and adopting mono-, di-, and tri-substituted carbazoles with tunable hole-transport ability as electron-donating groups. The developed hosts show the outstanding thermal stability with the *T*_d_ and *T*_m_ surpassing 350 °C and 200 °C, respectively. In addition, the absorption and emission spectra of the three compounds are highly similar, indicating the quantity of carbazoles does not change their intermolecular charge transfer feature. The high *E*_T_ levels of ~3.0 eV render these compounds suitable as the hosts for blue OLEDs. From **CzPhPS** to **TCzPhPS**, as the number of constructed carbazole units increases, the performance of their PhOLEDs becomes worse and worse with the maximum EQE decreasing from 17.5% to 7.2%, which can be ascribed to the deteriorated device’s charge transport balance. The current work is enlightening for designing bipolar hosts to achieve high-performance blue PhOLEDs.

## Figures and Tables

**Table 1:** Thermal, photophysical, electrochemical properties, and energy levels of the developed compounds.

Comp.	*T*_d_ [a]/*T*_m_ [b] (°C)	λ_abs_ [c] (nm)	λ_em_ [c] (nm)	PLQY [d] (%)	Lifetime [d] (ns)	HOMO (eV) Meas. [e]/ Calcd [f]	LUMO (eV) Meas. [g]/ Calcd [f]	*E*_g_ (eV) Meas. [h]/ Calcd [f]	*E*_T_ (eV) Meas. [i]/ Calcd [f]
**CzPhPS**	366/209	298, 332, 343	378	24	2.11	−5.71/−5.49	−2.29/−1.12	3.43/4.37	3.00/2.98
**DCzPhPS**	447/264	298, 335, 343	381	20	1.99	−5.70/−5.52	−2.28/−1.23	3.42/4.29	3.01/2.98
**TCzPhPS**	479/377	298, 335, 344	380	22	2.06	−5.74/−5.56	−2.34/−1.29	3.38/4.27	3.01/2.99

[a] Decomposition temperatures at 5 wt% weight loss; [b] Melting temperatures; [c] Absorption and PL peaks measured in solid thin films; [d] Measured in 10^−5^ M dichloromethane solution; [e] HOMO energy levels estimated from the onset potentials of the oxidation peaks in cyclic voltammograms; [f] Calculated from Gaussian 09 M062X; [g] Obtained from *E*_HOMO_ and *E*_g_ according to the equation of *E*_LUMO_ = *E*_g_ + *E*_HOMO_; [h] Estimated from the absorption edge of thin films (λ_onset_) employing the formula of *E*_g_ = 1240/λ_onset_; [i] Estimated from the first peaks of phosphorescence spectra recorded in tetrahydrofuran at 77 K.

**Table 2:** Electroluminescence (EL) performance of devices based on **CzPhPS**, **DCzPhPS**, and **TCzPhPS** hosts.

Device	Host	V_on_ [a] (°C)	λ [b] (nm)	Luminance [c] (cd m^−2^)	CE [d] (cd A^−1^)	PE [e] (lm W^−1^)	EQE [f] (%)
A	**CzPhPS**	3.1	472	17,223	36.7	37.5	17.5
B	**DCzPhPS**	3.4	472	17,176	32.3	29.8	16.1
C	**TCzPhPS**	3.4	476	12,500	15.4	14.2	7.2

[a] Turn-on voltage at a brightness of 1 cd m^−2^; [b] Electroluminescence peak wavelength at a voltage of 5 V; [c] Maximum luminance; [d] Maximum current efficiency; [e] Maximum power efficiency; [f] Maximum external quantum efficiency.

## Data Availability

The data presented in this study are available on request from the corresponding author.

## Supplementary Materials

The following are available online, Figures S1–S6: NMR spectra, Figures S7–S9: mass spectra, Figure S10: phosphorescence spectra.

## Sample Availability

Samples of **CzPhPS**, **DCzPhPS**, and **TCzPhPS** are available from the authors.
