# Supplementary material for: Phosphine Sulfide-Based Bipolar Host Materials for Blue Phosphorescent Organic Light-Emitting Diodes
Source: Molecules. 2021 Jul 3;26(13):4079. doi: 10.3390/molecules26134079 (PMC8271492; doi:10.3390/molecules26134079)
Supplement: Supplementary file 1 [file molecules-26-04079-s001.zip › molecules-1259802-supplementary.pdf]

Supplementary Materials

Phosphine Sulfide Based Bipolar Host Materials for Blue Phosphorescent Organic Light-Emitting Diodes

Gaozhan Xie[1], Jiangchao Wang[1], Yang Cao[1], Xudong Xue[1], Xiao Zhang[1], Chang Liu[1], Huanhuan Li[1,*], Ye Tao[1,*], and Runfeng Chen[1]

[1]State Key Laboratory of Organic Electronics and Information Displays & Institute of Advanced Materials (IAM), Jiangsu National Synergistic Innovation Center for Advanced Materials (SICAM), Nanjing University of Posts & Telecommunications, 9 Wenyuan Road, Nanjing 210023, China

*Correspondence: iamhhli@njupt.edu.cn and iamytao@njupt.edu.cn

Contents

1. NMR Spectra....................S2

2. Mass Spectra....................S5

3. Phosphorescence Spectra....................S6

4. Absorption and Photoluminescence Spectra....................S6

1. NMR Spectra

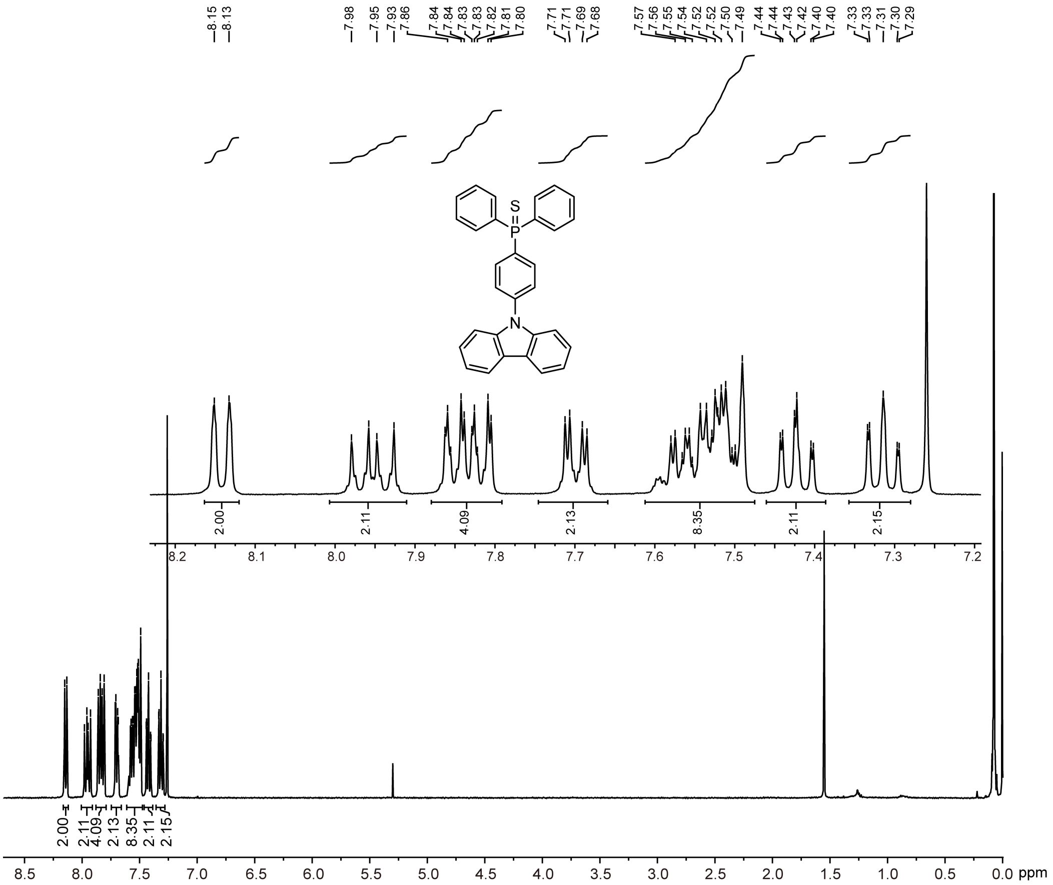

Figure S1. ^{1}H NMR spectrum of **CzPhPS** in $CDCl_3$.

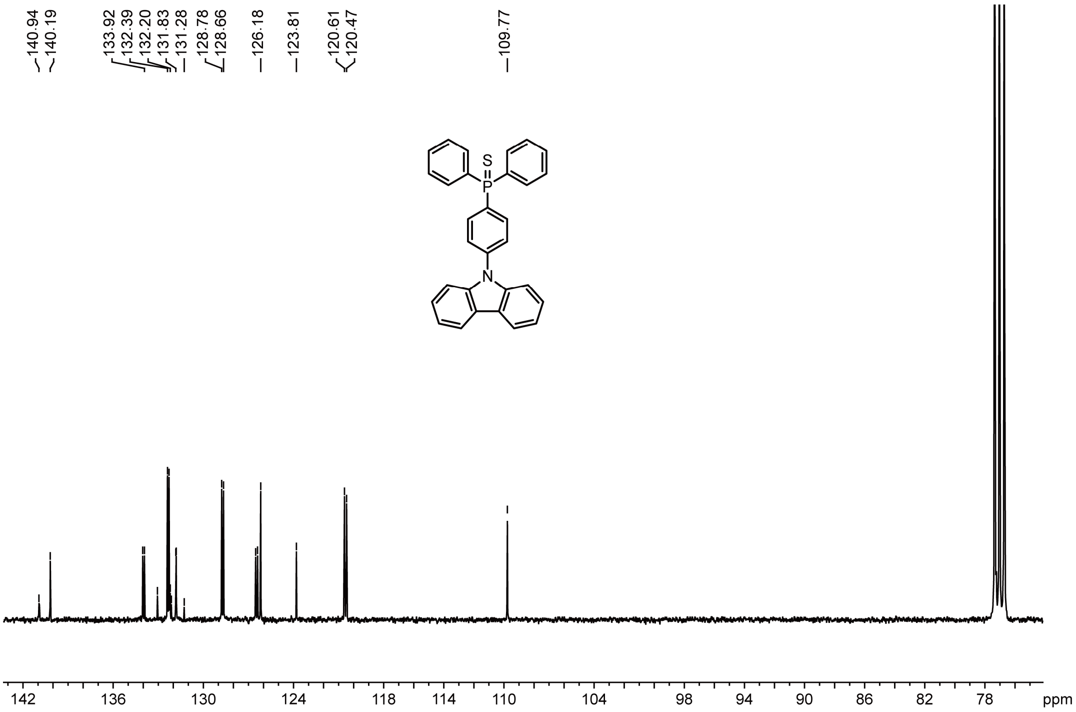

Figure S2. ^{13}C NMR spectrum of **CzPhPS** in $CDCl_3$.

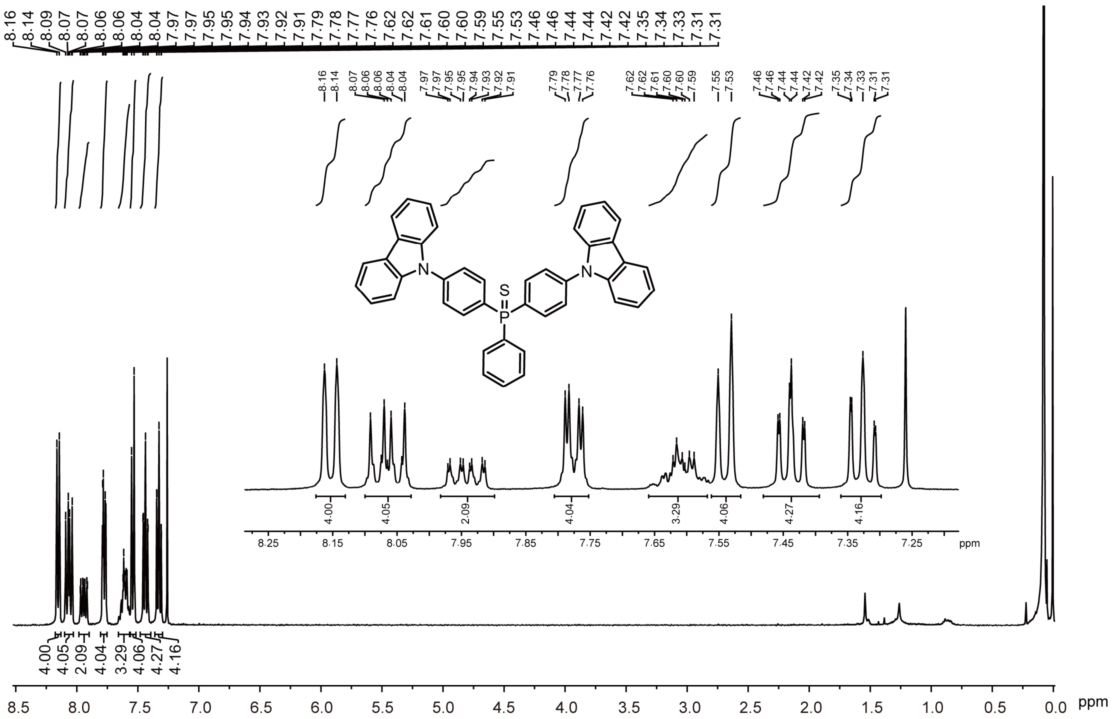

Figure S3. ^{1}H NMR spectrum of **DCzPhPS** in $CDCl_3$.

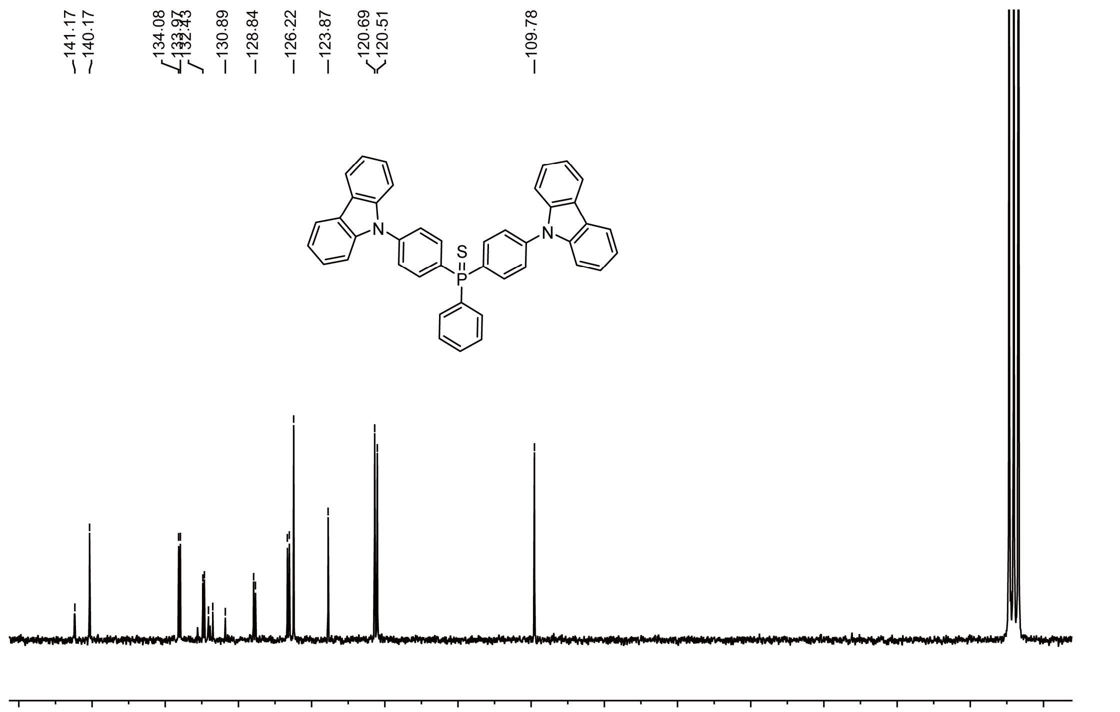

Figure S4. ^{13}C NMR spectrum of **DCzPhPS** in $CDCl_3$.

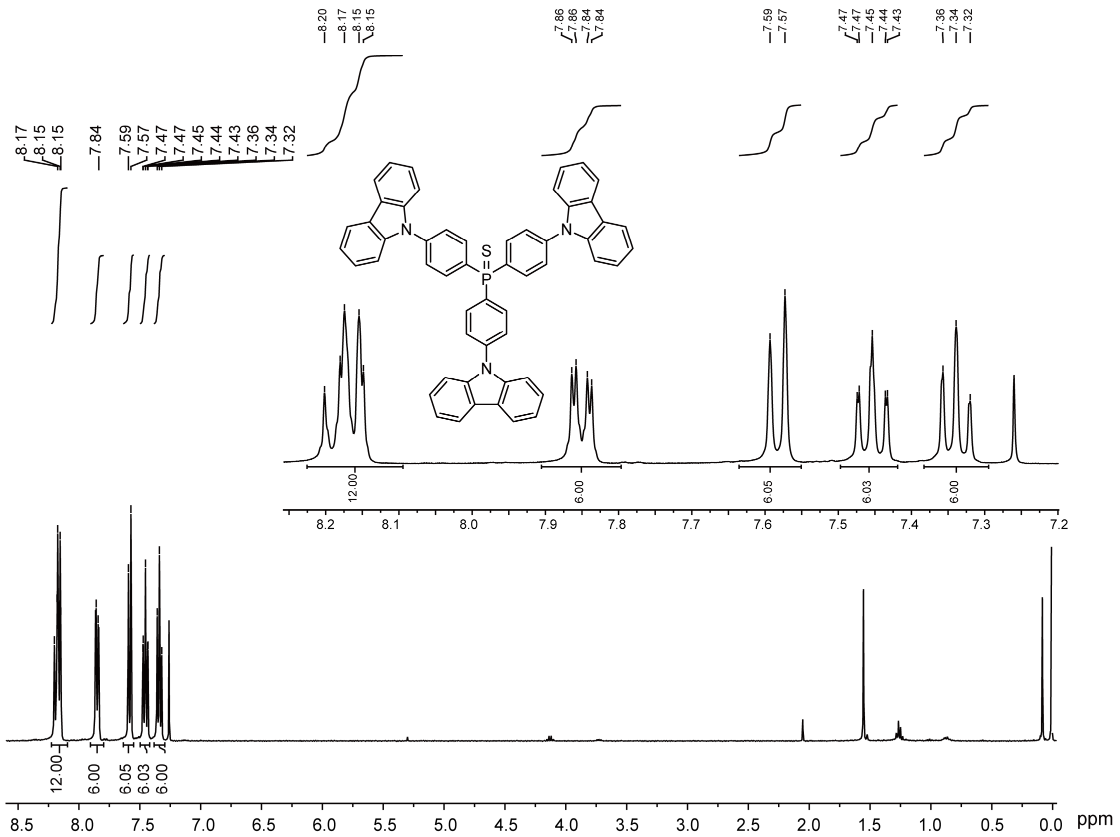

Figure S5. ^{1}H NMR spectrum of **TCzPhPS** in $CDCl_3$.

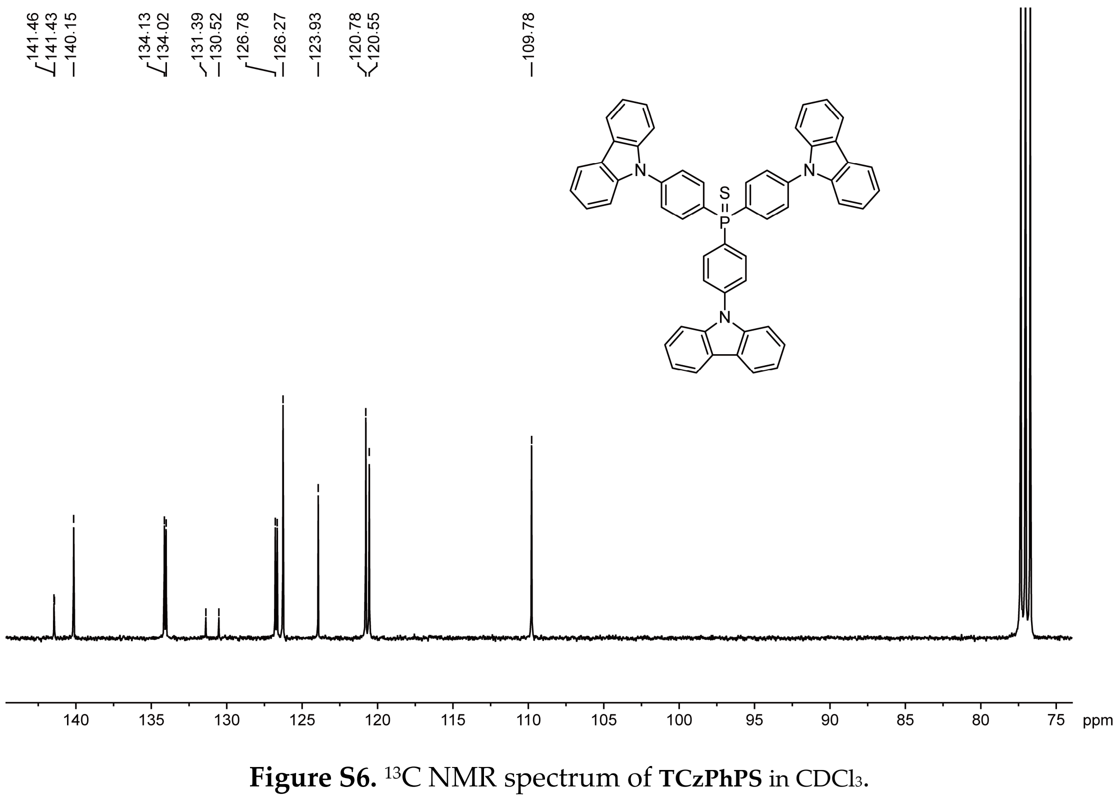

Figure S6. ^{13}C NMR spectrum of **TCzPhPS** in $CDCl_3$.

2. Mass Spectra

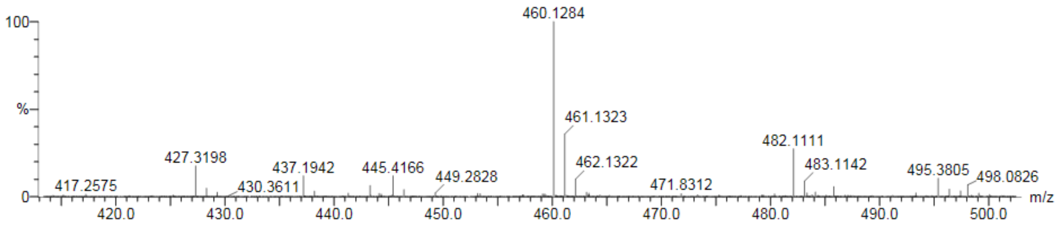

Figure S7. HRMS spectrum of **CzPhPS**.

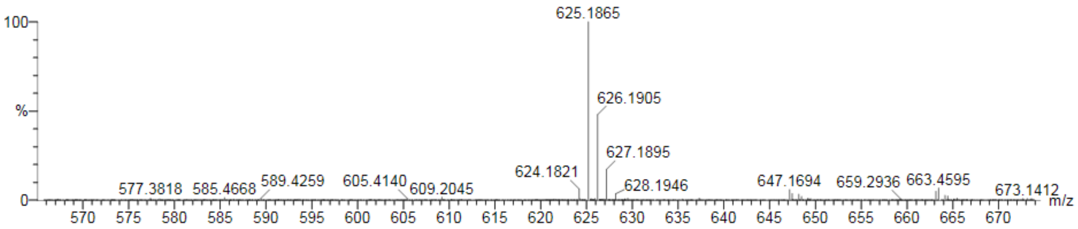

Figure S8. HRMS spectrum of **DCzPhPS**.

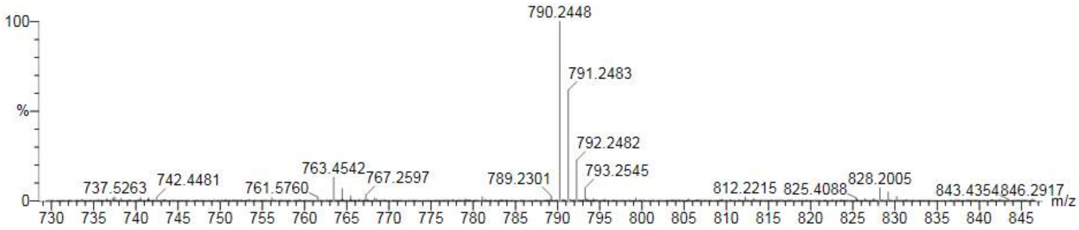

Figure S9. HRMS spectrum of **TCzPhPS**.

3. Phosphorescence Spectra

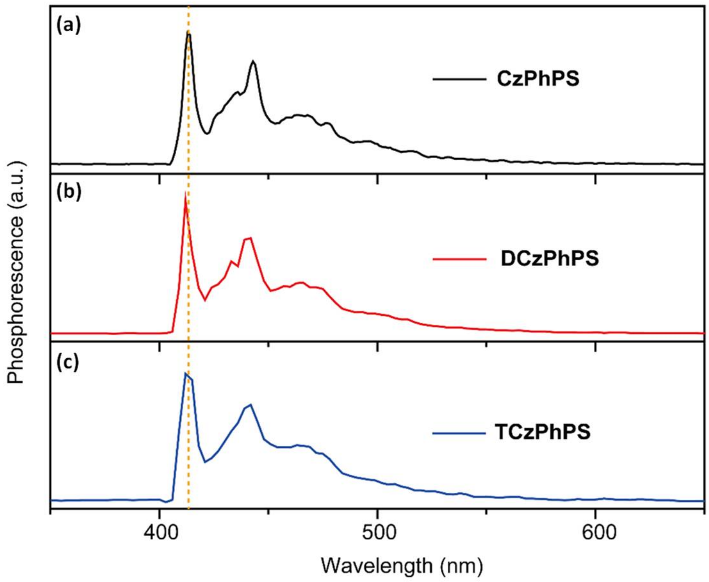

Figure S10. Phosphorescence spectra of (a) CzPhPS, (b) DCzPhPS, and (c) TCzPhPS measured at 77 K in THF with a delay time of 20 ms.

4. Absorption and Photoluminescence Spectra

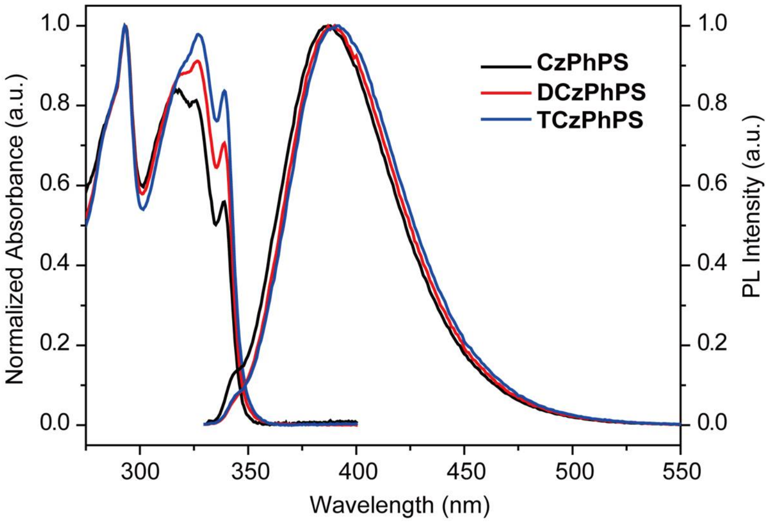

Figure S11. Absorption and PL spectra of **CzPhPS**, **DCzPhPS**, and **TCzPhPS** in dichloromethane solution**.**
